# Engineering A-Site Multi-Doping in Perovskite Oxide LaCoO_3_ for Tailored Radio-Frequency Dielectric Response and Electromagnetic Shielding Applications

**DOI:** 10.3390/ma19132916

**Published:** 2026-07-07

**Authors:** Tianze Wang, Chong Wang

**Affiliations:** 1 School of Materials Science and Chemical Engineering, Harbin Engineering University, Harbin 150001, China; 2 Department of Materials Science and Engineering, College of Transportation Engineering, Dalian Maritime University, Dalian 116026, China

**Keywords:** perovskite oxide, semiconductor, dielectric response, electromagnetic interference, (EMI) shielding

## Abstract

**Highlights:**

- A-site cation engineering enables effective regulation of radio-frequency dielectric responses in perovskite cobaltite oxides.
- Sr doping causes LaCoO_3_ to enter a metallic state, exhibiting a Drude-type dielectric behavior below the plasma frequency.
- Ternary La/Sr/Ba co-doping significantly reduces carrier concentration and plasma frequency, inducing a dielectric response transition.
- The symbolic change in dielectric response originates from a dominant mechanism transition from inductive to capacitive behavior.
- This work provides a material strategy for designing RF dielectric and electromagnetic functional materials.

**Abstract:**

The growing demand for high-performance electromagnetic interference (EMI) shielding materials in modern communication and integrated electronics has stimulated interest in materials with tunable dielectric responses. In this study, a series of A-site-doped perovskite oxides—LaCoO_3_, (La_0.5_Sr_0.5_)CoO_3_, and (La_1_/_3_Sr_1_/_3_Ba_1_/_3_)CoO_3_—were synthesized via a sol–gel method to investigate their dielectric behavior in the radio-frequency (RF) range. Dielectric spectroscopy reveals that LaCoO_3_ exhibits a positive permittivity characteristic of semiconductors, whereas Sr substitution induces a metallic state in (La_0.5_Sr_0.5_)CoO_3_, whose dielectric response exhibits a Drude-like dispersion behavior within the measured RF frequency range. Further incorporation of Ba into the A-site results in ternary co-doping, suggesting a reduction in effective carrier transport and a shift in the characteristic dispersion frequency toward the low-frequency region. Consequently, (La_1_/_3_Sr_1_/_3_Ba_1_/_3_)CoO_3_ displays a near-zero permittivity at approximately 2.5 kHz, indicating a transition in the dominant reactive response from inductive-like to capacitive-like behavior, which is consistent with the impedance spectroscopy results. This work demonstrates that cation engineering at the A-site enables precise control over the RF dielectric response in perovskite oxides, offering a potential pathway for the design of tunable electromagnetic functional materials relevant to EMI shielding applications with tailored permittivity characteristics.

## 1. Introduction

With the rapid advancement of integrated electronics and wireless communication systems, electromagnetic interference (EMI) has emerged as a critical challenge, necessitating the development of advanced materials with controllable electromagnetic properties [1,2,3,4]. Among these, dielectric response in the radio-frequency (RF) band plays a pivotal role in determining the electromagnetic wave absorption and reflection characteristics of materials, which are key to effective EMI shielding [5,6,7,8]. Among the various material parameters that influence shielding effectiveness, the dielectric response in the RF band plays a pivotal role in determining the electromagnetic wave absorption and reflection characteristics of materials. Specifically, the complex permittivity governs how an incident electromagnetic wave interacts with a shielding material: a high real part (ε′) enhances polarization and reflection, while a high imaginary part (ε″) contributes to dielectric loss and absorption. The ability to tailor these parameters independently is essential for designing shields that not only block unwanted signals but also minimize secondary reflections, which can cause additional interference in dense electronic environments. Traditional EMI shielding materials, such as metals and conductive polymers, often suffer from limited tunability, high density, or poor corrosion resistance, driving the search for alternative platforms. In this context, perovskite oxides with tunable dielectric responses offer a promising solution, as their electronic structures can be systematically modified via compositional engineering, defect control, or entropy stabilization. Achieving a precise balance between reflection and absorption, for instance, transitioning from positive permittivity to near-zero or even negative permittivity, enables impedance matching and enhanced wave attenuation, which are key to effective EMI shielding in next-generation wireless devices. Therefore, understanding and controlling the RF dielectric response of emerging material systems is not only a fundamental scientific pursuit but also a practical necessity for future electromagnetic compatibility and interference mitigation strategies.

Current strategies for tailoring dielectric responses can be broadly categorized into two approaches. The first involves artificially engineered metamaterials, where resonant structures are designed to achieve specific electromagnetic responses [9,10,11,12,13,14]. However, such systems often suffer from complex fabrication processes and limited integration potential for high-frequency and miniaturized applications [15,16,17,18]. The second approach relies on natural materials or their composites, where dielectric behavior is modulated through electronic structure engineering or microstructural design [19,20,21]. For instance, the incorporation of conductive fillers into insulating matrices can induce percolation behavior, enabling collective electron oscillations that give rise to Drude-type dielectric dispersion in the RF range [22,23,24].

Despite their versatility, composite systems face practical limitations, including susceptibility to oxidation, thermal instability, and parametric drift. These challenges have driven interest in single-phase functional materials, which offer enhanced chemical stability and greater controllability over electronic properties through doping and defect engineering [25,26,27,28]. For example, Drude-like dielectric responses have been observed in oxide systems such as Sr-doped LaMnO_3_ and oxygen-deficient indium tin oxide, demonstrating the feasibility of tuning plasma frequencies from the ultraviolet into the kHz–MHz regime [17,18]. Such advances highlight the promise of single-phase perovskites and related oxides as platform materials for designing low-frequency plasmonic shielding and reconfigurable RF devices. Looking forward, further exploration of entropy-stabilized single-phase systems could bridge the gap between fundamental dielectric physics and practical low frequency EMI shielding.

Among candidate materials, perovskite-type cobalt oxides, particularly LaCoO_3_, stand out due to their sensitivity to A-site cation substitution. Doping with divalent ions such as Sr^2+^ or Ba^2+^ not only alters the valence state of Co ions but also modulates carrier concentration and mobility, thereby enabling transitions from insulating to metallic behavior accompanied by significant changes in dielectric response [29]. Previous studies have shown that Ba-doped LaCoO_3_ can exhibit plasmon-like dielectric behavior in the kilohertz range [30], highlighting the potential of compositional engineering for low-frequency electromagnetic applications. By expanding the compositional space to ternary A-site co-doping (e.g., La, Sr, Ba), we can potentially achieve finer control over the plasma frequency, dielectric loss tangent, and impedance matching. This approach may enable the design of lightweight, corrosion-resistant, and thermally stable perovskite-based shielding materials capable of operating across a broader frequency spectrum. Furthermore, understanding the relationship between A-site disorder, electronic transport, and the inductive-capacitive transition is essential for developing next-generation reconfigurable RF devices and smart electromagnetic shields. Future research should systematically investigate how entropy-stabilized perovskite oxides can bridge the gap between low-frequency plasmonic behavior and low-frequency shielding demands.

However, most research has focused on binary doping systems, with limited exploration of how configurational entropy through multi-element co-doping affects the electronic structure and RF dielectric properties. Entropy engineering, which leverages configurational disorder to stabilize unique electronic states, has emerged as a promising paradigm in materials design [21,22,31]. Its application in perovskite oxides remains largely unexplored, particularly in the context of electromagnetic shielding. For example, high-entropy alloys have shown exceptional strength and corrosion resistance, while high-entropy oxides have exhibited unusual ionic conductivity and dielectric anomalies. Despite these advances, the application of entropy engineering to perovskite oxides especially in the context of electromagnetic interference (EMI) shielding remains largely unexplored. Most existing studies on perovskite-based shielding materials still rely on conventional single- or binary-doped compositions, leaving the vast compositional space of medium- to high-entropy perovskites virtually untapped. This knowledge gap is particularly critical because electromagnetic shielding at RF frequencies demands materials with precisely tunable complex permittivity, low reflection loss, and high absorption efficiency properties that could potentially be optimized through entropy-driven electronic structure modulation. Therefore, systematically investigating how configurational entropy affects the RF dielectric response of perovskite oxides, and establishing the structure–property relationships that govern their shielding performance, represents a timely and highly promising research direction. Filling this gap will not only deepen fundamental understanding of entropy-stabilized electronic phenomena but also accelerate the development of next-generation adaptive shielding materials for future wireless communication systems.

In this work, we systematically investigate the effects of A-site multi-doping on the radio-frequency (RF) dielectric response of LaCoO_3_-based perovskites, with a particular focus on their potential for electromagnetic interference (EMI) shielding. By designing compositions that span from low-entropy LaCoO_3_ to medium-entropy (La_0.5_Sr_0.5_)CoO_3_ and finally to (La_1_/_3_Sr_1_/_3_Ba_1_/_3_)CoO_3_, we demonstrate a remarkable transition in dielectric behavior: from positive permittivity (typical of semiconductors) to Drude-type dispersion, and ultimately to a near-zero permittivity state. This composition-driven evolution is interpreted through the Drude model and complex impedance analysis, revealing the underlying mechanism of the inductive-to-capacitive transition. The key innovation of this work lies in the use of ternary A-site co-doping to achieve medium-entropy stabilization, which enables fine control over carrier concentration and plasma frequency—a strategy rarely explored for RF shielding applications. From a research significance perspective, this work provides a preliminary material platform for exploring RF dielectric regulation in perovskite oxides where tunable permittivity is essential for achieving optimal impedance matching and effective wave attenuation. Unlike conventional shielding materials that rely solely on reflection, our perovskite system allows dynamic adjustment of both reflection and absorption mechanisms by simply varying the compositional entropy. This offers a pathway toward lightweight, adaptive shielding solutions capable of addressing complex interference environments. Looking forward, the integration of such compositionally engineered perovskites into next-generation wireless communication systems including Internet of Things (IoT) devices, and smart electromagnetic environments holds great promise. Potential applications include reconfigurable shielding films, frequency-selective surfaces, and tunable RF absorbers. Future research should focus on optimizing doping stoichiometry for broader bandwidth shielding effectiveness, exploring multilayer architectures, and developing flexible thin-film deposition techniques. Ultimately, this work not only advances fundamental understanding of dielectric responses in perovskites but also opens new avenues for intelligent, high-performance electromagnetic shielding materials essential for future high-frequency electronics.

## 2. Materials and Methods

### 2.1. Chemicals

In this work, lanthanum nitrate hexahydrate (purity ≥ 98.5%), strontium nitrate (purity ≥ 99.5%), barium nitrate (purity ≥ 99.5%), cobalt Nitrate Hexahydrate (purity ≥ 98.5%), ethylene glycol (purity ≥ 99.5%), and citric acid monohydrate (purity ≥ 99.8%) were purchased from Sinopharm Chemical Reagent Co., Ltd. (Shanghai, China).

### 2.2. Synthesis of Ceramics

The LaCoO_3_, (La_0.5_Sr_0.5_)CoO_3_, and (La_1_/_3_Sr_1_/_3_Ba_1_/_3_)CoO_3_ ceramics were synthesized via a sol–gel method followed by a solid-state reaction process.

**Figure 1:**
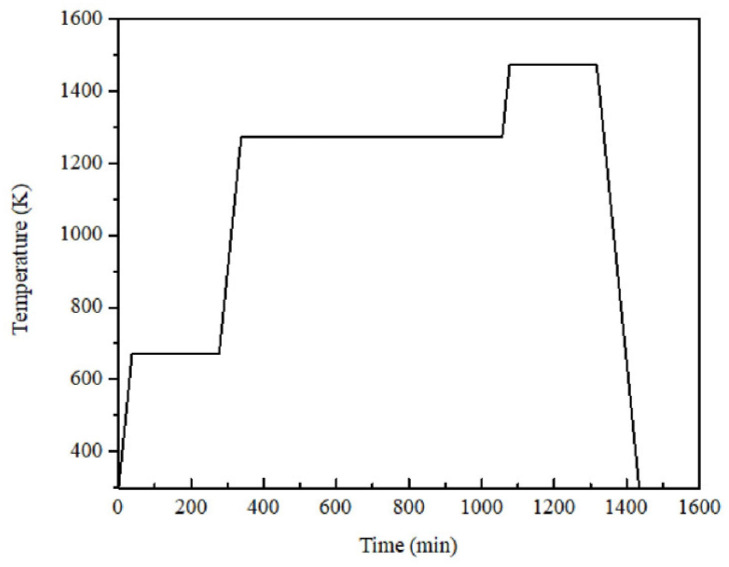
The sintering process.

LaCoO_3_: Stoichiometric amounts of La(NO_3_)_3_·6H_2_O (1 mmol) and Co(NO_3_)_2_·xH_2_O (1 mmol) were dissolved in deionized water to form a clear nitrate solution. After stirring for 30 min, citric acid was added as a chelating agent in a molar ratio of 2:1 relative to the total metal cations, corresponding to 4 mmol. Subsequently, ethylene glycol was introduced, with a volume ratio of 1:20, into the aqueous solution. The mixture was heated at 353 K under constant stirring until a viscous gel was obtained upon solvent evaporation. The resulting gel was then heated in a furnace at 473 K for 4 h in air to form a loose precursor. This precursor was ground into a fine powder and subjected to a calcination treatment at 1273 K for 12 h in air to form the crystalline LaCoO_3_ phase. The powder was uniaxially pressed into pellets and finally sintered in a muffle furnace at 1473 K for 4 h in air to obtain dense ceramics. Figure 1 shows the sintering process.

(La_0.5_Sr_0.5_)CoO_3_: The synthesis procedure was identical to that used for LaCoO_3_, except for the initial reagents. La(NO_3_)_3_·6H_2_O (1 mmol), Sr(NO_3_)_2_ (1 mmol), and Co(NO_3_)_2_·xH_2_O (2 mmol) were used as starting materials. The amount of citric acid was accordingly set to 8 mmol. The obtained precursor was calcined at 1273 K for 12 h to form the (La_0.5_Sr_0.5_)CoO_3_ phase, and the powder was sintered at 1473 K for 4 h.

(La_1_/_3_Sr_1_/_3_Ba_1_/_3_)CoO_3_: This compound was prepared using a similar protocol. Stoichiometric proportions of La(NO_3_)_3_·6H_2_O (1 mmol), Sr(NO_3_)_2_ (1 mmol), Ba(NO_3_)_2_ (1 mmol), and Co(NO_3_)_2_·xH_2_O (3 mmol) were dissolved in deionized water. A total of 12 mmol of citric acid was added. The subsequent steps, including gel formation, precursor calcination at 1273 K, and final sintering of the pellets at 1473 K for 4 h, were consistently applied to yield the final (La_1_/_3_Sr_1_/_3_Ba_1_/_3_)CoO_3_ ceramics.

### 2.3. Characterization and Measurement

The phase structure of the ceramics was characterized by X-ray diffraction (XRD, Rigaku D/MaxB, Akishima-shi, Tokyo, Japan). Microstructural analysis was performed using a field emission scanning electron microscope (FESEM, Philips XL-30, Eindhoven, Netherlands). The dielectric properties, including the real permittivity (*ε*′) and imaginary permittivity (*ε*″), as well as the complex impedance (*Z*′ and *Z*″), were measured in the frequency range of 1 kHz to 1 MHz using an impedance analyzer (Agilent 4294A, Kobe, Japan). During the test, the powder samples were pressed into thin sheets with a thickness of 2 mm and a diameter of 10 mm. Then, a pair of parallel plate electrodes were used to place the samples in contact for the test. Thus, all the test conditions were the same. To minimize geometric and contact-related variations, all samples were measured under identical testing conditions, including pellet dimensions, electrode configuration, and measurement parameters. Nevertheless, considering the relatively low-frequency range (1 kHz–1 MHz) and the conductive nature of some samples, contributions from electrode polarization, interfacial charge accumulation, and conductivity-related dielectric artifacts cannot be completely excluded. Therefore, the dielectric response discussed in this work was interpreted primarily from a phenomenological perspective.

## 3. Results and Discussion

### 3.1. The Synthesis and Characterization of (La_1/3_Sr_1/3_Ba_1/3_)CoO_3_

**Figure 2:**
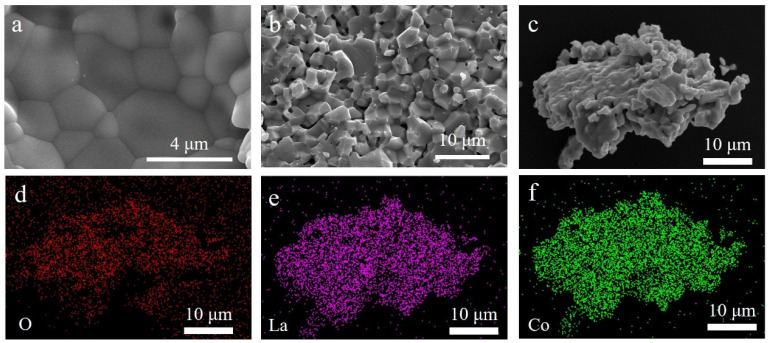
(**a**–**c**) SEM image of LaCoO_3_. (**d**–**f**) SEM-Mapping image of LaCoO_3_ with O, La and Co elements.

Scanning electron microscopy (SEM) was used to analyze the morphology of the synthesized LaCoO_3_. As shown in Figure 2a,b, a clear lattice structure and grain boundaries can be observed from the microscopic morphology of LaCoO_3_, indicating that the synthesized LaCoO_3_ has strong crystallinity. In addition, energy-dispersive X-ray spectroscopy (EDS) was also used for surface scanning elemental analysis of LaCoO_3_, as shown in Figure 2c–f, and Figure 2d–f is the corresponding scanning image of O, La, and Co elements. As shown in the figure, O, La and Co elements are uniformly distributed in the synthesized LaCoO_3_ sample.

**Figure 3:**
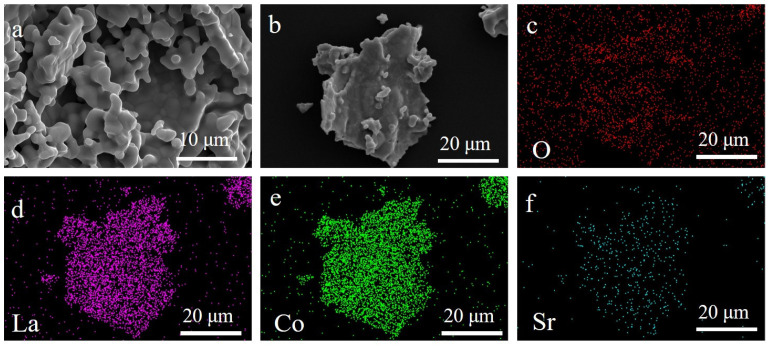
(**a**,**b**) SEM image of (La_0.5_Sr_0.5_) CoO_3_. (**c**–**f**) SEM mapping of (La_0.5_Sr_0.5_)CoO_3_ with O, La, Co and Sr elements.

SEM was employed to examine the morphology of the (La_0.5_Sr_0.5_)CoO_3_ ceramics. As clearly shown in Figure 3a,b, the material retains well-defined lattice fringes and discernible grain boundaries. Despite the introduction of Sr atoms into the lattice, which substitute for some of the La sites, the sample maintains a high degree of crystallinity. To further investigate the elemental distribution, EDS mapping was performed, with the results displayed in Figure 3d–f. The corresponding maps for O, La, Co, and Sr reveal that all four elements are uniformly distributed throughout the scanned area. Oxygen is abundant, as expected, while La, Co, and Sr also exhibit homogeneous dispersion without noticeable aggregation, confirming a chemically homogeneous sample at the microscale.

**Figure 4:**
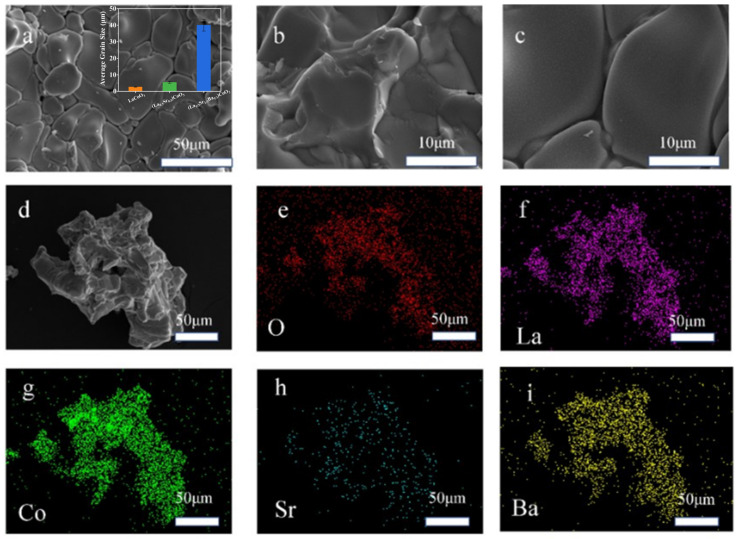
(**a**–**d**) SEM image of (La_1/3_Sr_1/3_Ba_1/3_)CoO_3_. The illustration shows the distribution of grain sizes of the sample. (**e**–**i**) SEM mapping of (La_1/3_Sr_1/3_Ba_1/3_)CoO_3_ with O, La, Co, Sr and Ba.

The microstructure of the as-synthesized (La_1_/_3_Sr_1_/_3_Ba_1_/_3_)CoO_3_ was investigated by SEM, as shown in Figure 4a–d. The images reveal well-defined lattice fringes and clear grain boundaries, albeit with an increased surface roughness compared to the binary counterparts. This observation indicates that the increased cationic complexity at the A-site did not disrupt the fundamental perovskite crystal structure, and the material retained its highly crystalline nature. The grain sizes were counted and are shown in the inset of Figure 4a. It is obvious that as the doping at the A site increases, the grain sizes become larger. Furthermore, elemental distribution was analyzed by EDS mapping, with the results displayed in Figure 4e–i. The corresponding maps for O (e), La (f), Co (g), Sr (h), and Ba (i) demonstrate a homogeneous distribution of all five elements within the scanned region, confirming a chemically uniform sample at the microscale. The quantitative confirmation of elemental composition from EDS analysis was provided in Figure S1.

**Figure 5:**
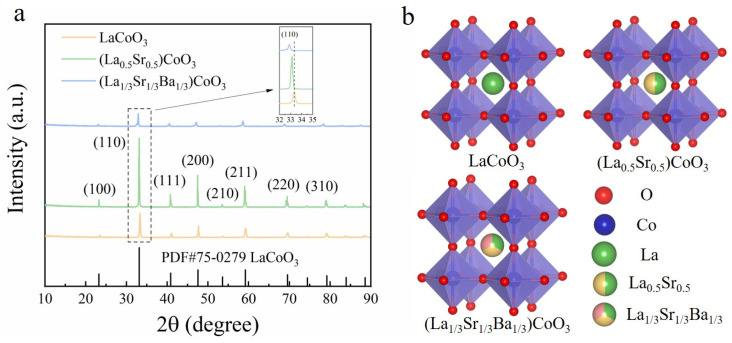
(**a**) XRD patterns of LaCoO_3_ (orange line), (La_0.5_Sr_0.5_)CoO_3_ (green line), and (La_1/3_Sr_1/3_Ba_1/3_)CoO_3_ (blue line). (**b**) The crystal structures of three materials.

The crystal structures of the as-synthesized LaCoO_3_, (La_0.5_Sr_0.5_)CoO_3_, and (La_1_/_3_Sr_1_/_3_Ba_1_/_3_)CoO_3_ samples were examined by X-ray diffraction (XRD). As presented in Figure 5a, all the diffraction peaks for LaCoO_3_ can be well indexed to the rhombohedral perovskite structure (JCPDS PDF#75-0279), with observed peaks at 2θ values of 23.3°, 33.1°, 40.9°, 47.6°, 53.6°, 59.2°, 69.5°, and 79.2°, corresponding to the (100), (110), (111), (200), (210), (211), (220), and (310) crystal planes, respectively. The absence of any impurity peaks confirms the successful synthesis of a pure-phase LaCoO_3_. Furthermore, the XRD patterns of both (La_0.5_Sr_0.5_)CoO_3_ and (La_1_/_3_Sr_1_/_3_Ba_1_/_3_)CoO_3_ exhibit a similar set of diffraction peaks. This indicates that the A-site substitution with Sr and Ba does not alter the fundamental perovskite framework, thereby verifying the successful formation of the target doped compounds without secondary phases. The peak intensity of (La_0.5_Sr_0.5_)CoO_3_ is the highest because the A sites are composed of La^3+^ and Sr^2+^ in a 1:1 ratio, and the ionic radii match well, making it easy to form a uniform solid solution in the perovskite structure. The peak intensity of LaCoO_3_ is the second lowest. This is because although its crystal structure is stable, the grain size of this material is relatively small. The crystalline peak intensity of (La_1_/_3_Sr_1_/_3_Ba_1_/_3_)CoO_3_ is the lowest because Ba^2+^ with a larger ionic radius is introduced at the A site, making ion diffusion and rearrangement more difficult. Under the same sintering conditions, the crystallization is incomplete. The diffraction peaks of the (110) crystal planes systematically shift towards lower angles (to the left) with the doping at the A site. The radius of the A-site doping ions is La^3+^ < Sr^2+^ < Ba^2+^, which leads to an increase in the average radius of the A site successively. This is the primary reason for the systematic left shift of the peak positions. Using the XRD data, we calculated the d-spacing of (110): 2.337, 2.366, and 2.396 Å for LaCoO_3_, (La_0.5_Sr_0.5_)CoO_3_, and (La_1_/_3_Sr_1_/_3_Ba_1_/_3_)CoO_3_. The clear increase in d-spacing with increasing A-site doping (from mono-doping to multi-doping) confirms a systematic lattice expansion, which is consistent with the larger average ionic radii of the substituted cations (Sr^2+^ with 1.44 Å, Ba^2+^ with 1.61 Å) compared to La^3+^ (1.36 Å) for 12-coordination. Figure 5b shows the crystal structures of three materials.

### 3.2. The Dielectric Performance

**Figure 6:**
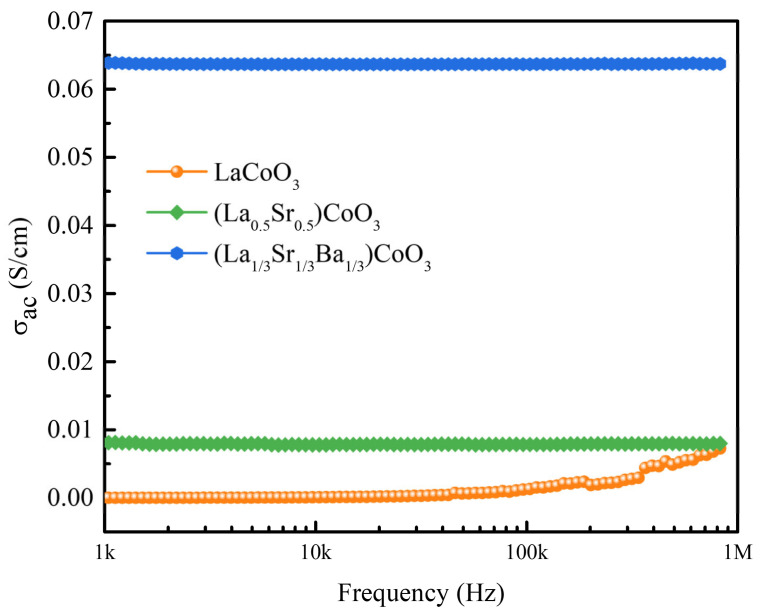
The *σ*_ac_ spectra of LaCoO_3_, (La_0.5_Sr_0.5_)CoO_3_, and (La_1/3_Sr_1/3_Ba_1/3_)CoO_3_.

Figure 6 demonstrates the alternating current conductivity (*σ*_ac_) spectra of LaCoO_3_, (La_0.5_Sr_0.5_)CoO_3_, and (La_1/3_Sr_1/3_Ba_1/3_)CoO_3_. As shown in the figure, pristine LaCoO_3_ exhibits an upward trend with the increase in frequency, revealing that hopping conductivity dominates the charge transport. In contrast, for the (La_0.5_Sr_0.5_)CoO_3_ and (La_1/3_Sr_1/3_Ba_1/3_)CoO_3_, the *σ*_ac_ remains nearly unchanged as the frequency increases, suggesting the transition of direct current-like conductivity. Meanwhile, the incorporation of cationic elements (Sr and Sr, Ba) significantly elevates the overall magnitude of *σ*_ac_ of the perovskite oxide system.

**Figure 7:**
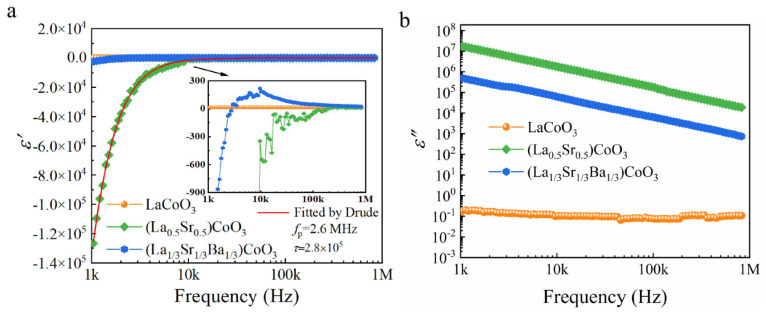
(**a**) Diagram of the relationship between the real part (*ε*′) of the permittivity and frequency from 1 kHz to 1 MHz at room temperature. The illustration is an enlarged view with the vertic al axis ranging from −900 to 300. (**b**) Diagram of the relationship between the imaginary part (*ε*″) of permittivity and frequency from 1 kHz to 1 MHz at room temperature.

Figure 7a shows the frequency dependence of the real part (*ε*′) of the complex permittivity of the synthesized sample. LaCoO_3_ exhibits a positive *ε*′ value throughout the entire measurement frequency range (1 kHz–1 MHz), which is a typical characteristic of semiconductor materials [32]. The existing band gap prevents electrons from being excited to the conduction band by the low-energy electromagnetic field in this frequency band. Therefore, the dielectric response is mainly dominated by the electron skip conductance, presenting a positive permittivity.

In contrast, the *ε*′ value of (La_0.5_Sr_0.5_)CoO_3_ shows significant dispersion behavior within the same frequency band. Sr replacing half of the La sites alters the electronic structure of the material, significantly enhancing its electrical conductivity and carrier transport behavior. In this metal-like state, free electrons will undergo collective oscillation under the influence of an external electromagnetic field, resulting in a dielectric dispersion behavior qualitatively resembling the classical Drude-type response [33]:

(1) $$\varepsilon_{r}^{\prime }\left(\omega \right)=1-\frac{\omega_{p}^{2}}{\omega^{2}+\omega_{\tau }^{2}}$$

(2) $$\omega_{p}=\sqrt{\frac{{e^{2}n}_{eff\;}}{m_{eff}\varepsilon_{0}}}$$

where *ω_p_* (*ω_p_* = 2π*f_p_*) represents the angular plasma frequency, *f_p_* is the plasma frequency, *ω* (*ω* = 2π*f*) indicates the angular frequency of the applied electromagnetic field, *ω_τ_* (*ω_τ_* = 2π*f_τ_*) stands for the damping parameter, *τ* is the relaxation time, ε_0_ is the permittivity of vacuum (8.85 × 10^−12^ F/m), n_eff_ indicates the effective concentration of conduction electrons, *m_eff_* represents the effective weight of electron and *e* stands for the electron charge (1.6 × 10^−19^ C). The sign and magnitude of *ε*′ are essentially related to the plasma frequency (*f*_p_) of the material. When the measurement frequency is lower than the plasma frequency, *ε*′ exhibits the dispersion characteristics predicted by the Drude model. Since the plasma frequency is directly proportional to the square root of the carrier density, it can be effectively regulated through chemical doping. The increase in entropy caused by co-doping may influence carrier transport dynamics and thereby shift the plasma frequency towards lower frequencies, which may be related to a decrease in carrier concentration or an increase in relaxation time. And the experimental result was fitted using the Drude model, with *R*^2^ value of 0.99, indicating the goodness of fit.

This regulatory principle is reflected in (La_1_/_3_Sr_1_/_3_Ba_1_/_3_)CoO_3_. The introduction of Ba^2+^ with a larger ionic radius effectively reduces the carrier concentration, thereby lowering its plasma frequency to the kHz range. A dielectric crossover behavior can be observed around approximately 2.5 kHz, with the value approaching a minimum (about 0). The occurrence of ε′ approaching zero suggests a balance between conductive and polarization-related contributions to the dielectric response, which has been extensively studied for applications such as wave tunneling, directive emission, and cloaking. In the present system, this crossover frequency may be associated with the characteristic dispersion frequency of charge transport. Table 1 shows the comparison of permittivity value for (La_1_/_3_Sr_1_/_3_Ba_1_/_3_)CoO_3_ and other dielectric materials [21,34,35,36]. Figure 7b shows the frequency dependence of dielectric loss (*ε*″). As shown in the figure, LaCoO_3_ has the lowest dielectric loss, while (La_0.5_Sr_0.5_)CoO_3_ has the highest loss, which is consistent with their respective semiconductor and conductor characteristics [37].

**Figure 8:**
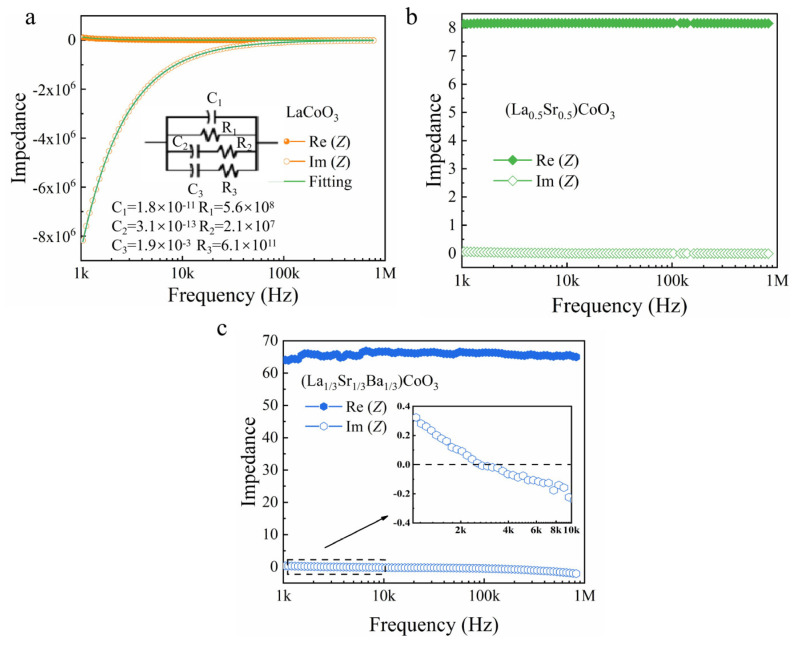
(**a**–**c**) Impedance real part (Re (*Z*)) and imaginary part (Im (*Z*)) data of LaCoO_3_, (La_0.5_Sr_0.5_)CoO_3_ and (La_1/3_Sr_1/3_Ba_1/3_)CoO_3_ from 1 kHz to 1 MHz at room temperature.

To further clarify the intrinsic mechanism of the dielectric response, we measured the complex impedance. Impedance real part (Re (*Z*)) and imaginary part (Im (*Z*)) represent the resistance and reactance. Figure 8a shows the impedance frequency spectrum of LaCoO_3_. The Im (*Z*) is negative in the range of 1 kHz to 1 MHz, indicating that its dominant mechanism is capacitive, which is consistent with the observed positive permittivity response. The impedance-based equivalent circuit model was used to analyze its mechanism. The equivalent circuit including resistors and capacitors was constructed as shown in the figure. The results obtained by fitting with the equivalent circuit were in good agreement with the experimental data, which suggests that the equivalent circuit can reasonably describe the measured impedance behavior within the investigated frequency range. On the contrary, (La_0.5_Sr_0.5_)CoO_3_ exhibits a positive Im (*Z*) value within the same frequency band (Figure 8b), which suggests the presence of inductive-like reactive behavior that is consistent with conductive carrier transport characteristics. The impedance response evolution of (La_1_/_3_Sr_1_/_3_Ba_1_/_3_)CoO_3_ is clearly visible (Figure 8c). Below 2.5 kHz, a positive Im (*Z*) value confirms that inductive behavior is dominant. However, above 2.5 kHz, Im (*Z*) turns negative, indicating that the dominant mechanism transforms into capacitive behavior. This evolution of the reactance behavior indicates a frequency-dependent transition in the dominant electrical response mechanism [38]. The transition near ~2.5 kHz corresponds to the frequency where the real permittivity *ε*′ crosses zero (from positive to negative). In the Drude model, this occurs when *ω* = *ω*_p_ (plasma frequency). Below plasma frequency, the inductive (free-carrier) response dominates, giving *ε*′ < 0; above plasma frequency, the capacitive (bound-electron) response dominates, giving *ε*′ > 0. Therefore, this transition may be associated with a plasma-like dielectric crossover behavior.

## 4. Conclusions

This study demonstrates that the ternary A-site co-doping strategy incorporating La, Sr, and Ba into the perovskite LaCoO_3_ enables precise modulation of carrier concentration and electronic structure. This approach successfully transforms the radio-frequency (RF) dielectric response from a typical semiconductor-like positive behavior into a frequency-dependent response featuring a sign reversal—a dielectric characteristic potentially relevant to electromagnetic functional materials. The observed transition is qualitatively consistent with a plasma-like dielectric dispersion behavior. The physical origin of this transition is unequivocally linked to the tuning of the plasma frequency, which is phenomenologically interpretable using the Drude model and further corroborated by comprehensive impedance spectroscopy analysis. The resulting compound, (La_1_/_3_Sr_1_/_3_Ba_1_/_3_)CoO_3_, exhibits a compositionally tunable dielectric response, making it highly promising for impedance matching in RF circuits and reconfigurable RF devices. From an electromagnetic shielding perspective, this material may offer potential advantages: the ability to dynamically adjust its complex permittivity enables both reflection and absorption mechanisms to be tailored, thereby addressing the growing demand for lightweight, adaptive shielding solutions against unwanted EM interference. Looking forward, the integration of such compositionally engineered perovskites into next-generation wireless communication systems, particularly 6G networks, Internet of Things (IoT) ecosystems, and smart electromagnetic environments, may provide prospective insights for the design of reconfigurable shields and frequency-selective surfaces. Future research should focus on optimizing the doping stoichiometry for enhanced shielding effectiveness across broader bandwidths, exploring multilayer architectures, and developing thin-film deposition techniques compatible with flexible substrates. Ultimately, this work not only provides a new material platform for fundamental dielectric studies but also opens a novel pathway toward intelligent, adaptive electromagnetic shielding solutions essential for future high-frequency electronics [39,40,41].

## Figures and Tables

**Table 1:** The comparison of permittivity value for (La_1_/_3_Sr_1_/_3_Ba_1_/_3_)CoO_3_ and other dielectric materials.

Samples	Frequency Range	Permittivity Value	References
La_0.84_Ba_0.16_CO_3_	1 MHz	~−5000	[34]
Cu/PVDF	1 MHz	~−10,000	[35]
BaTiO_3_/Cu	1 GHz	~−6000	[36]
BaTiO_3_/Ni	900 MHz	0	[21]
(La_1_/_3_Sr_1_/_3_Ba_1_/_3_)CoO_3_	~2.5 kHz	0	This work

## Data Availability

The original contributions presented in this study are included in the article/Supplementary Materials. Further inquiries can be directed to the corresponding author.

## Supplementary Materials

The following supporting information can be downloaded at https://www.mdpi.com/article/10.3390/ma19132916/s1. Figure S1: EDS elemental composition of (A) LaCoO_3_, (B) (La_0.5_Sr_0.5_) CoO_3_ and (C) (La_1/3_Sr_1/3_Ba_1/3_)CoO_3_.

## Abbreviations

The following abbreviations are used in this manuscript:

EMI	electromagnetic interference
RF	radio-frequency
XRD	X-ray diffraction
SEM	scanning electron microscope
EDS	energy-dispersive X-ray spectroscopy
