# Supplementary material for: Engineering A-Site Multi-Doping in Perovskite Oxide LaCoO3 for Tailored Radio-Frequency Dielectric Response and Electromagnetic Shielding Applications
Source: Materials (Basel). 2026 Jul 7;19(13):2916. doi: 10.3390/ma19132916 (PMC13363364; doi:10.3390/ma19132916)
Supplement: Supplementary file 1 [file materials-19-02916-s001.zip › materials-4372847-supplementary.pdf]

materials

MDPI

Article

Engineering A-Site Multi-Doping in Perovskite Oxide $LaCoO_3$ for Tailored Radio-Frequency Dielectric Response and Electromagnetic Shielding Applications

Tianze Wang [1] and Chong Wang [2,*]

[1] School of Materials Science and Chemical Engineering, Harbin Engineering University, Harbin 150001, China
[2] Department of Materials Science and Engineering, College of Transportation Engineering, Dalian Maritime University, Dalian 116026, China
* Correspondence: wangchong0218@dlmu.edu.cn

Supplementary Materials

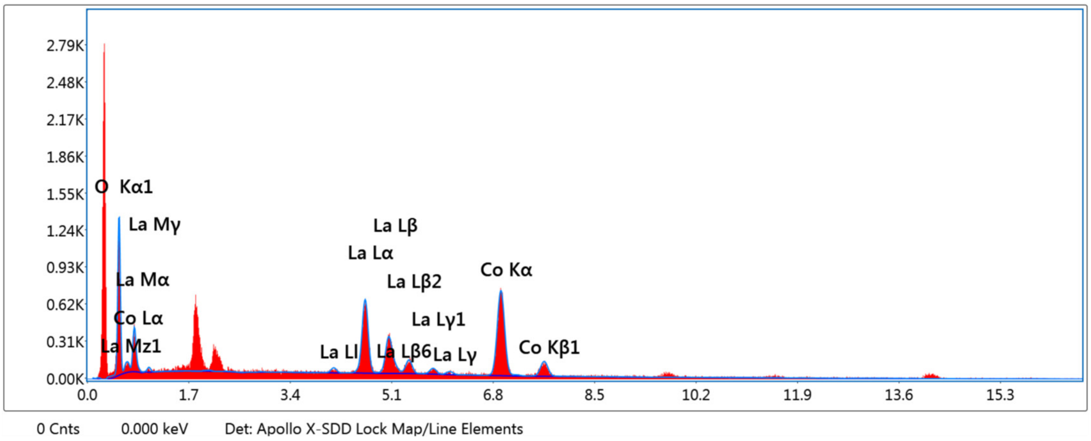

Smart Quant Results

Element	Weight %	Atomic %	Net Int.	Kratio	Z	A	F
O K	33.1	71.9	35.2	0.1749	1.2146	0.4345	1.0000
LaL	33.2	8.3	38.5	0.3042	0.8016	1.1321	1.0097
CoK	33.7	19.8	53.8	0.2895	0.9563	0.8893	1.0110

Elemental composition of $LaCoO_3$.

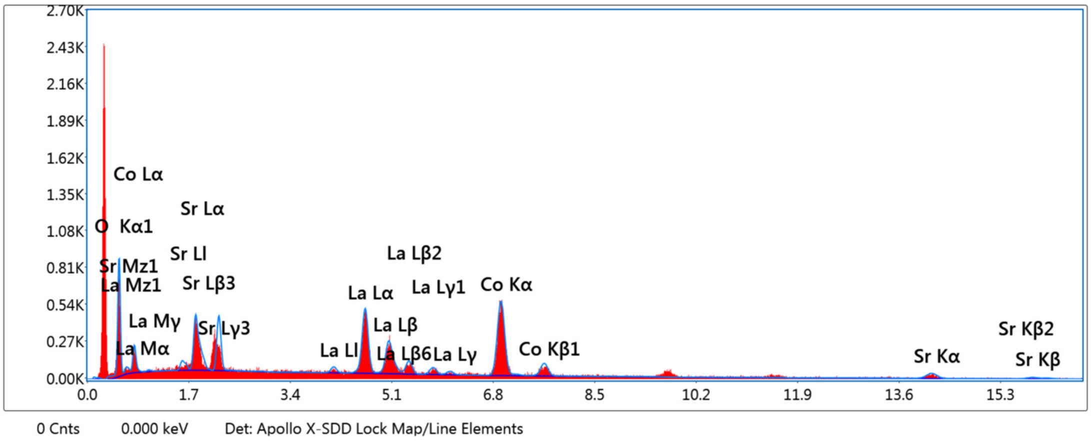

Smart Quant Results

Element	Weight %	Atomic %	Net Int.	Kratio	Z	A	F
O K	32.9	72.3	22.9	0.0957	1.2179	0.2389	1.0000
LaL	22.7	5.8	29.9	0.1990	0.8038	1.0826	1.0052
CoK	21.2	12.6	41.6	0.1885	0.9591	0.9060	1.0251
SrK	23.2	9.3	4.2	0.2146	0.8520	0.9915	1.0943

Elemental composition of (La$_{0.5}$Sr$_{0.5}$) CoO$_3$.

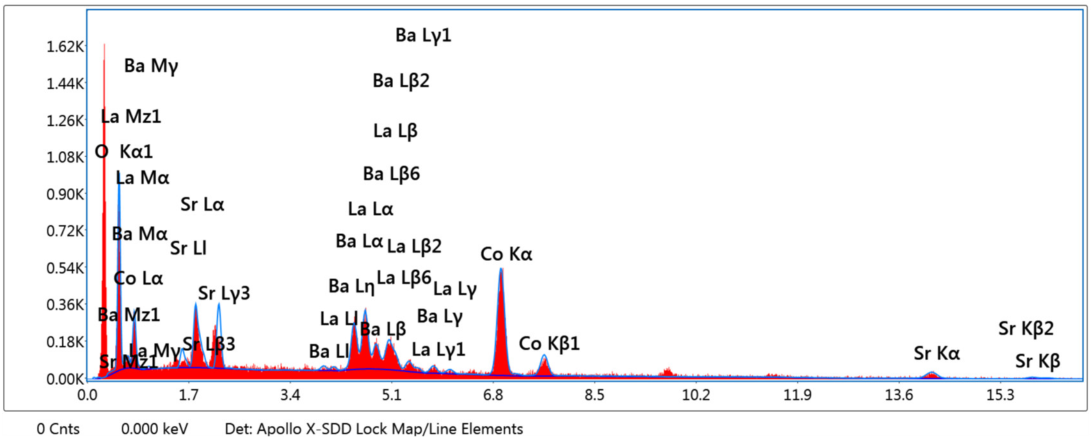

Smart Quant Results

Element	Weight %	Atomic %	Net Int.	Kratio	Z	A	F
O K	34.4	73.9	25.9	0.1112	1.2139	0.2665	1.0000
BaL	11.6	2.9	14.0	0.1009	0.7979	1.0843	1.0068
LaL	13.8	3.4	17.8	0.1212	0.8011	1.0887	1.0053
CoK	21.1	12.3	40.1	0.1865	0.9558	0.9032	1.0235
SrK	19.1	7.5	3.4	0.1782	0.8487	0.9912	1.1097

(C) Elemental composition of (La$_{1/3}$Sr$_{1/3}$Ba$_{1/3}$)CoO$_3$.

Figure S1. EDS elemental composition of (A) $LaCoO_3$, (B) (La$_{0.5}$Sr$_{0.5}$) CoO$_3$ and (C) (La$_{1/3}$Sr$_{1/3}$Ba$_{1/3}$)CoO$_3$.
